# Sleep and physical activity trade-offs and dementia risk: a prospective cohort study in UK Biobank participants

**DOI:** 10.1186/s12916-025-04536-7

**Published:** 2025-12-03

**Authors:** Stephanie Yiallourou, Lachlan Cribb, Beaudan Campbell-Brown, Christian Brakenridge, Andree-Ann Baril, Matthew P. Pase

**Affiliations:** 1 Turner Institute for Brain and Mental Health & School of Psychological Sciences, Monash University, 18 Innovation Walk, Room 617, Melbourne, 3800 Australia; 2 School of Health Sciences, Swinburne University of Technology, Melbourne, 3122 Australia; 3 Baker Heart & Diabetes Institute, 75 Commercial Rd, Melbourne, 3004 Australia; 4 Center for Advanced Research in Sleep Medicine, Hôpital du Sacré-Coeur de Montréal, Recherche CIUSSS NIM, 5400 Boul Gouin O, Montreal, QC H4J 1C5 Canada; 5 Department of Medicine, Université de Montréal, Pavillon Roger-Gaudry, 2900 Edouard Montpetit Blvd, Montreal, QC H3T 1J4 Canada

**Keywords:** 24-h behaviors, Sleep, Physical activity, Sedentary behavior, Dementia, Compositional analysis, Accelerometry, Brain volumes, MRI

## Abstract

**Background:**

Engaging in regular physical activity and obtaining recommended amounts of sleep are touted as strategies to promote healthy brain aging. However, as each day is only 24 h long, changing time spent in one activity must come at the expense or gain of another, making it necessary to understand how the whole 24-h activity composition is associated with dementia risk. We investigated the effect of substituting sleep duration for different levels of physical activity (i.e., inactivity, light activity, and moderate to vigorous physical activity; MVPA) in short sleepers (< 6 h) and normal sleepers (≥ 6 h and ≤ 9 h).

**Methods:**

The study sample comprised 87,490 participants from the community-based UK Biobank, with 24-h behaviors estimated using up to 7 days of accelerometry. Participants were free from dementia or severe neurological disease at baseline. The main outcome was the risk of incident all-cause dementia over a median follow-up of 8.2 years.

**Results:**

The mean age of the sample was 63 years (Q1, Q3, 56, 68); 56% were women.

For short sleepers, increasing sleep duration was associated with a lowering of dementia risk when at the expense of inactivity or light activity, but not when at the expense of MVPA. For normal sleepers, the effect of increasing or decreasing sleep duration on dementia risk differed for all three substituted behaviors (i.e., inactivity, light, or MVPA). Most notably, increasing sleep at the expense of MVPA was associated with greater dementia risk, and increasing MVPA at the expense of sleep was associated with lower dementia risk. The interpretation of the results was broadly consistent when using MRI-based outcomes (e.g., hippocampal volume) in a subset with brain imaging (*n* = 15,180).

**Conclusions:**

Our findings from this observational analysis suggest that personalized approaches that balance trade-offs between sleep duration and differing physical activity levels based on individual circumstances, such as habitual sleep duration, may be important for dementia risk reduction.

**Supplementary Information:**

The online version contains supplementary material available at 10.1186/s12916-025-04536-7.

## Background

Sleep may have a functional role in the clearance of amyloid beta and tau proteins, which can aggregate to form the two pathological hallmarks of Alzheimer’s disease (AD) [1, 2]. Both short (≤ 6 h) [3] and long (> 9 h) [4] sleep durations have been associated with an increased risk of dementia [5]. Daytime activity levels have also been linked to dementia risk. For example, low physical activity levels and high sedentary time are associated with higher dementia risk [6, 7]. Intuitively, normalizing sleep duration or increasing physical activity could be attractive targets to reduce dementia risk. However, as each day is only 24 h long, changing time spent in one activity must come at the expense or gain of another. Consequently, the potential benefits of several lifestyle interventions are not straightforward; their efficacy may depend on the substituted behavior. For example, although increasing physical activity in the form of exercise or sport has been touted as one strategy to lower dementia risk, is it advisable if it comes at the expense of sleep time? Current recommendations do not define what type of behavior should be replaced when increasing time spent in physical activity [8].

Robust dementia prevention guidelines involving sleep duration or daytime physical activity levels (i.e., sedentary time, light physical activity, and moderate to vigorous physical activity; MVPA) require a complete understanding of how adding and subtracting time spent in different activities affects dementia risk. Furthermore, there is a need to understand the effect of adding and subtracting sleep duration for varying levels of physical activity (or inactivity), given the role of sleep in glymphatic clearance and that the benefits of physical activity on cognitive health may be dependent on the amount of sleep [9]. Previous studies on sleep time and physical activity’s link to dementia have tended to overlook the fact that these activities are a quantitative component of a 24-h composition. Traditional regression methods isolate sleep or daytime activities with only partial adjustment for time spent in other behaviors, ignoring that time-use behaviors are co-dependent (if one behavior increases, another must decrease).

To overcome these limitations, compositional data analysis (CoDA) can be applied to examine 24-h behaviors and dementia risk. Unlike standard regression tools, CoDA respects the fact that time-use data are constrained to 24 h and allows for estimating the effect of explicit time-use substitutions (e.g., adding 30 min of sleep at the expense of 30 min of MVPA). A study of 1899 participants in the Rotterdam cohort examined 24-h time-use substitutions and found that spending more time in MVPA and less in other behaviors was associated with a lower risk of dementia [10]. Similarly, reallocating time from sedentary or light activity to sleep was linked to reduced dementia risk. However, this study was limited by its relatively small sample and short follow-up time (4.5 years). In contrast, a much larger study of 484,169 UK Biobank participants applied CoDA to investigate time-use substitutions and dementia incidence, reporting that replacing sedentary time with physical activity reduced dementia risk [11]. This analysis, however, relied on self-reported measures and excluded sleep, focusing only on daytime sedentary and physical activity behaviors. To date, no large-scale studies with long follow-up have investigated time-use substitutions across the full 24-h day using objective measures.

We applied CoDA to a large community-based cohort with 24-h behaviors estimated through up to 7 days of accelerometry monitoring and dementia follow-up. We aimed to determine (1) the association between substituting sleep duration for daytime physical activities (inactivity, light activity, and MVPA) on dementia risk and MRI volumetric outcomes and (2) the “lowest risk” composition of 24-h behaviors (i.e., the absolute time spent in sleep, inactivity, light activity, and MVPA) associated with the lowest estimated dementia risk in these data.

## Methods

### Study design and participants

This prospective cohort study uses data from the UK Biobank (UKB) (access date 21/03/2024). The UK Biobank recruited over 500,000 UK adults aged 40–69 years during 2006–2010 [12]. Detailed lifestyle, environmental, and genetic details were collected at the baseline session (described in detail on the study website: https://www.ukbiobank.ac.uk/). Follow-up for the occurrence of several health outcomes is ongoing. Participants identified as living within a reasonable traveling distance of the 22 UKB assessment centers in the UK National Health Service patient registers were invited by mail to participate in the study. The participants who agreed to participate tended to be older, had higher socioeconomic status (SES), and were more likely to be female than non-participants, with a participation fraction of 5.5% [13]. Between February 2013 and December 2015, 236,519 UKB participants were invited to participate in a 7-day wrist-worn accelerometer study. A total of 106,053 agreed to take part [14]. For this study, participants with dementia or other severe neurological diseases (Parkinson’s disease, motor neuron disease, cerebral palsy, brain abscess, multiple sclerosis, or myasthenia gravis) at or before the time of the accelerometry study were excluded. The sample selection process from the total UKB sample is displayed in Additional file 1, Figure S1 [3, 15–18].

### Consent statement.

All UKB participants provided informed consent to participate in the study. UK Biobank has approval from the North West Multi-Centre Research Ethics Committee as a Research Tissue Bank (RTB) approval. This approval means the present study operates under the RTB approval, satisfying the requirements of the Monash University Human Research Ethics Committee. The study was performed in accordance with the ethical standards as laid down in the 1964 Declaration of Helsinki and its later amendments or comparable ethical standards.

### Measurement of 24-h behaviors by accelerometry

For the objective assessment of 24-h behaviors, participants underwent 7 days and nights of wrist-worn accelerometry (Axivity AX3). Most participants (88%) provided complete accelerometry data. Further details of the accelerometry protocol have been published previously [14]. For the determination of time spent in sleep, inactivity, light activity, and MVPA, we used the open-source R package GGIR v2.7–1 [19]. Raw acceleration data were processed using the Euclidean Distance Minus One procedure (ENMO) [15]. The default GGIR thresholds were used to distinguish inactivity from light activity and MVPA: < 30 mg for inactivity, ≥ 30 and < 100 mg for light, and ≥ 100 mg for MVPA. As a sleep diary was not available in the UKB, time in sleep was estimated using the algorithm of van Hees et al. [16, 17]. Sleep duration was captured from the main sleep period time window, the longest block of contiguous sleep within a 24-h period, which excludes periods of sustained inactivity bouts (reflecting naps/inactivity) during waking hours.

Low-quality accelerometer data were removed using the established UKB criteria: lack of agreement between self-reported wear time and accelerometer wear time data (5%); insufficient wear time (< 72 h; 5%); and poor calibration (< 1%). Additionally, data were removed for participants for whom GGIR was unable to determine a sleep window (5%) and for participants with time spent in each behavior below the 0.1st or greater than the 99.9th percentile (to limit the influence of extreme values and possible data errors; < 1%).

### Dementia case ascertainment

Incident all-cause dementia was ascertained based on hospital records, death records, and primary care [20]. As UKB record linkage with primary care providers is ongoing, primary care record linkage was only available for slightly less than half of the complete sample (approximately 45%). The algorithms used to ascertain all-cause dementia were designed to maximize positive predictive value, which has been shown to be high (> 80% for each record type) [21]. The list of Read V2 codes (primary care) and ICD-10 codes informing the identification of all-cause dementia has been published previously [22].

### Brain MRI outcomes

The UKB imaging sub-study began in 2014 and aimed to acquire high-quality and consistent imaging data from 100,000 UKB participants across multiple modalities. All MRI data were acquired on 3 T Siemens Skyra scanners. Imaging-derived phenotypes (IDPs) were derived using an automated pipeline described in depth previously [23]. Outcomes used for the current study included total brain volume, hippocampal volume, total grey matter volume, total white matter volume, and white matter hyperintensity volume. Volumetric IDPs of interest were normalized for head size using a head-size scaling factor [24].

### Data analysis

Average daily time spent in each of the four behaviors (sleep, inactivity, light, MVPA) was averaged across the accelerometry wear days and standardized over the day of the week to ensure comparability of those with and without weekend assessments. As compositional time-use variables cannot be included in traditional statistical models, such as multivariable regression, due to collinearity among components [25], these were transformed from the simplex into the Real space using an isometric log ratio (ILR) transformation [26].

Confounder variables were selected using a directed acyclic graph (Additional file 1, Figure S2). Primary model covariates included age, sex, education, ethnicity, *APOE ε4* genotype, household income, fruit and vegetable intake, alcohol intake, antidepressant, antipsychotic, or sedative medication use, retirement status, and shift work. Missing data in confounder variables were infrequent (most < 2%) and were imputed by predictive mean matching using the R package *mice* [27]*.* One imputed dataset was created per bootstrap sample (see below). Imputation models included all confounder, exposure, and outcome variables.

### Isotemporal substitution

To investigate isotemporal substitutions, we first fitted parametric pooled logistic regression models [18], including the ILR transformed compositional variables and covariates, to estimate the hazards of dementia and death for each individual in the sample. The pooled logistic models included restricted cubic splines for continuous predictors, product terms between the exposures and time, between exposures and key covariates, and between key covariates and time. Time from baseline was used as the time scale.

We then split the data into short sleeper (< 6 h per night) and normal sleeper subgroups (≥ 6 h and ≤ 9 h per night) for our primary analysis. We selected these sleep duration cut-offs because (1) prior studies have shown that short sleep (< 6 h) (3) and long sleep (> 9 h) (4) are associated with increased dementia risk, and (2) both < 6 and > 9 h fall outside the expert consensus recommendations for optimal sleep to support overall, cognitive, emotional, and physical health in adults [28]. In each subgroup, we estimated the cumulative incidence of dementia (hereafter, “risk”) by the end of follow-up under the observed composition (i.e., without applying any substitution) using the gformula estimator of the “total effect” described by Young et al. [29]. These risks represented the risk under no intervention on time use and provided a reference point for investigating isotemporal substitutions. Next, for each individual in the dataset, we modified their observed time use composition by adding up to 1 h (in 15-min steps) to a given behaviour and subtracting it from another behaviour, such as adding 30 min to sleep and subtracting 30 min from MVPA. These substitutions were only applied to an individual so long as the composition resulting from that substitution was within the range of the sample data. If the resulting composition for an individual was outside of this range, the substitution was not applied for them. If fewer than 75% of the subgroup was able to receive a given substitution, risk ratios for this substitution were not presented. Dementia risk under each substituted composition and dementia risk ratios, relative to the reference described above, were estimated. As a secondary analysis, we also considered a long sleeper subgroup. We chose > 8 h as the threshold for long sleep as very few participants (< 0.1%) had > 9 h sleep. See eMethods for details. Nonparametric bootstrapping was used to obtain percentile-based 95% confidence intervals.

### Lowest risk, typical, and highest risk compositions

To estimate the “lowest risk” composition, the time-use pattern associated with the lowest estimated dementia risk, we used the following algorithm: first, we created a grid of synthetic compositions (in 15-min steps) covering all possible compositions within the range of the sample data, excluding those estimated to be rare or implausible (see eMethods). We then split the data into two equally sized folds. In the first (“training”) data fold, dementia risk was estimated under each of the synthetic compositions using the parametric g-formula approach described above. The compositions returning the lowest and highest estimated risks were deemed the “lowest risk” and “highest risk” compositions, respectively. The “typical” composition was the synthetic composition estimated to be the most common (see eMethods). Next, in the second (“testing”) data fold, the cumulative incidence of dementia was estimated as above for each of the three compositions. We used this cross-validation procedure to ensure that different subsets of the data were used for identifying and evaluating the lowest and highest risk compositions, thereby providing protection against overoptimism. The algorithm is described in greater detail in the eMethods.

### MRI volumetric outcomes

A linear regression model was fitted for volumetric MRI outcomes including covariates and the ILR coordinates as predictors. Following UKB recommendations, these models were additionally adjusted for the MRI assessment center, mean fMRI head motion, and head location in the scanner [24]. The mean difference in volumetric MRI outcomes for a given substitution was estimated by g-computation, analogously to the dementia models. Additionally, estimated volumetric MRI outcomes were estimated for the “lowest risk”, “typical”, and “highest risk” compositions that we calculated in the previous step (for dementia risk).

### Sensitivity analyses

In a first sensitivity analysis, we included additional adjustment for sleep fragmentation (wake after sleep onset), also assessed by accelerometry. In a second, we additionally adjusted for covariates that may be plausibly affected by time use, including history of cardiovascular disease, diabetic status, body mass index, blood pressure medication, systolic blood pressure, and sickness or disability (obtained from the self-reported employment variable). In a third, we removed participants with dementia events during the first three years of follow-up. A substantial change in the estimated risk ratios in this sensitivity analysis may indicate reverse causation. Finally, to assess the effect of selection bias due to the non-representativeness of the UKB (healthy cohort effect), we standardized to the distributions of sex, retirement status, income, and smoking status of the representative UKB pseudo-population described by Schoeler et al. [30].

## Results

Characteristics of the sample at baseline are described in Table 1. The total sample size was 87,490. The mean age of the sample at the time of accelerometry was 63 years (Q1, Q3, 56, 68); 56% were women. There were 718 incident all-cause dementia cases over a median follow-up of 8.2 years (25th percentile 7.6; 75th percentile 8.7). Of the dementia cases, 97% (*n* = 671) were diagnosed after age 60. The median age of dementia occurrence was 76 years. The median time between accelerometry and MRI assessments was 3.4 years (25th percentile 1.9; 75th percentile 4.4). Results are presented separately for the normal sleepers (≥ 6 h and ≤ 9 h, *n* = 69,589 [80%]), short sleepers (< 6 h, *n* = 17,885 [20%]), and long sleepers (> 8 h, *n* = 2,178 [2.5%]).

**Table 1:** Baseline sample characteristics

Characteristic	Summary
Age, years, median (Q1, Q3)	63 (56, 68)
Women, *n* (%)	49,877 (56)
BMI, kg/m^2^, median (Q1, Q3)	26.0 (23.6, 29.0)
Highest qualification, *n* (%)	
High school non-completers	12,988 (15)
High school completers	5476 (6.2)
Trade qualification	10,162 (12)
Graduate degree	51,799 (59)
Other	7332 (8.3)
Prefer not to answer	373 (0.4)
Employment, *n* (%)	
Paid employment	54,836 (62)
Retired	27,628 (31)
Sick or disabled	1341 (1.5)
Other	4652 (5.2)
Prefer not to answer	159 (0.2)
Shift work, *n* (%)	3870 (4.4)
Average total household income (thousand pounds), *n* (%)	
< 18	11,642 (13)
18–30	19,221 (22)
31–50	22,902 (26)
52–100	19,989 (23)
> 100	5800 (6.6)
Don’t know/prefer not to answer	8479 (9.6)
Ethnicity, *n* (%)	
White	81,571 (92)
Asian	3424 (3.9)
Other	3371 (3.8)
Prefer not to answer	250 (0.3)
Antidepressant medication	5009 (5.7)
Insomnia medication	749 (0.8)
Smoking status, *n* (%)	
Never	50,561 (57)
Former	31,780 (36)
Current	6076 (6.9)
Daily alcohol intake, *n* (%)	20,029 (23%)
Townsend deprivation index, median (Q1, Q3)	− 3.8 (− 2.4, − 0.2)
*APOE ε4* alleles, *n* (%)	
0	53,357 (72)
1	19,150 (26)
2	1629 (2.2)
History of cancer, *n* (%)	11,503 (13)
History of cardiovascular disease, *n* (%)	35,789 (41)
History of diabetes, *n* (%)	3689 (4.2)
Average sleep duration, hours/day*	6.5 (1.2)
Average inactivity, hours/day*	12.0 (1.1)
Average light activity, hours/day*	3.3 (1.3)
Average moderate to vigorous activity, hours/day*	1.7 (1.5)

^*^Geometric mean (geometric standard deviation)

### Time-use substitutions for normal sleepers

**Fig. 1:**
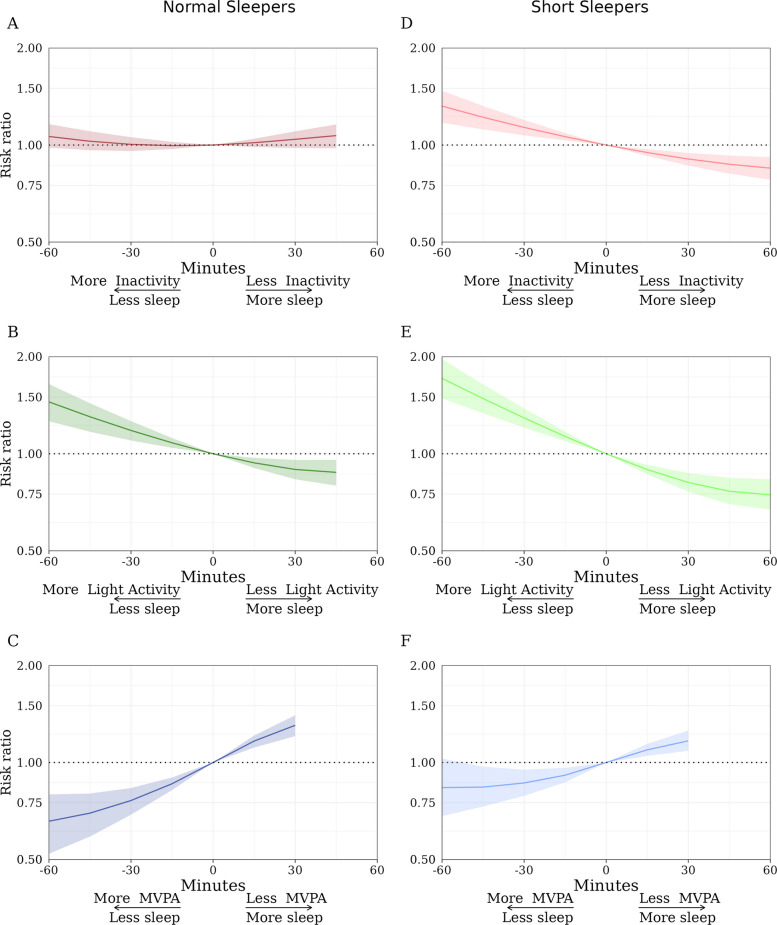
All-cause dementia risk ratio (and 95% confidence interval) for time-use substitutions for normal (**a**, **b**, and **c**) and short sleepers (**d**, **e**, and **f**). MVPA = moderate to vigorous physical activity. Normal sleepers are defined as persons with ≥ 6 h and ≤ 9 h of sleep (0.1% of the sample had > 9 h of sleep). Short sleepers are defined as persons with < 6 h of sleep

Dementia risk ratios for time-use substitutions in 15-min intervals, for normal sleepers are presented in Fig. 1a–c and Additional File 1, Table S1. Replacing inactivity with sleep time (and vice versa) had little association with dementia risk (Fig. 1a). In contrast, replacing light activity with sleep time was associated with a lowering of dementia risk (Fig. 1b); the RR associated with reallocating 30 min/day of light activity to sleep was 0.89 (95% CI, 0.83, 0.96), while the RR associated with reallocating 30 min/day of sleep to light activity was 1.18 (95% CI, 1.10, 1.26). Replacing MVPA with sleep time was associated with the largest increase in dementia risk (Fig. 1c); the RR associated with reallocating 30 min/day of MVPA to sleep was 1.30. (95% CI, 1.21, 1.40), while the RR associated with reallocating 30 min/day of sleep to MVPA was 0.76 (95% CI, 0.69, 0.83). Thus, increasing or decreasing sleep duration had a different association with dementia risk for all three substituted behaviors.

### Time-use substitutions for short sleepers

Dementia risk ratios for time-use substitutions in 15-min intervals for short sleepers are presented in Fig. 1d–f and Additional File 1, Table S1. Unlike in normal sleepers, replacing inactivity with sleep in short sleepers was associated with a lowering of dementia risk (Fig. 1d); the RR associated with reallocating 30 min/day of inactivity to sleep was 0.91 (95% CI, 0.86, 0.95), while the RR associated with reallocating 30 min/day of sleep to inactivity was 1.13 (95% CI, 1.07, 1.20). In short sleepers, substitutions involving sleep and light activity, and sleep and MVPA appeared broadly consistent with the results observed for normal sleepers, except for one crucial difference: whereas increasing MVPA at the expense of sleep was associated with a lower risk of dementia in normal sleepers, this was attenuated in short sleepers (Fig. 1f). The RR associated with reallocating 30 min/day of sleep to MVPA was 0.86 (95% CI, 0.79, 0.95). The RR associated with reallocating 30 min/day of MVPA to sleep was 1.17 (95% CI, 1.09, 1.26). The largest dementia risk in short sleepers was observed when replacing sleep with light activity; the RR associated with reallocating 30 min/day of sleep to light activity was 1.29 (95% CI, 1.21, 1.38; Fig. 1e). Overall, for short sleepers, increasing sleep duration was associated with a lowering of dementia risk when at the expense of inactivity or light activity, but not MVPA.

### Time-use substitutions for long sleepers

Dementia risk ratios for time-use substitutions for long sleepers are displayed in Additional file 1, Figure S3. For long sleepers, reallocating time from sleep to other behaviors was associated with reduced risk in all cases except for light activity.

### Highest risk, typical, and lowest risk compositions

**Fig. 2:**
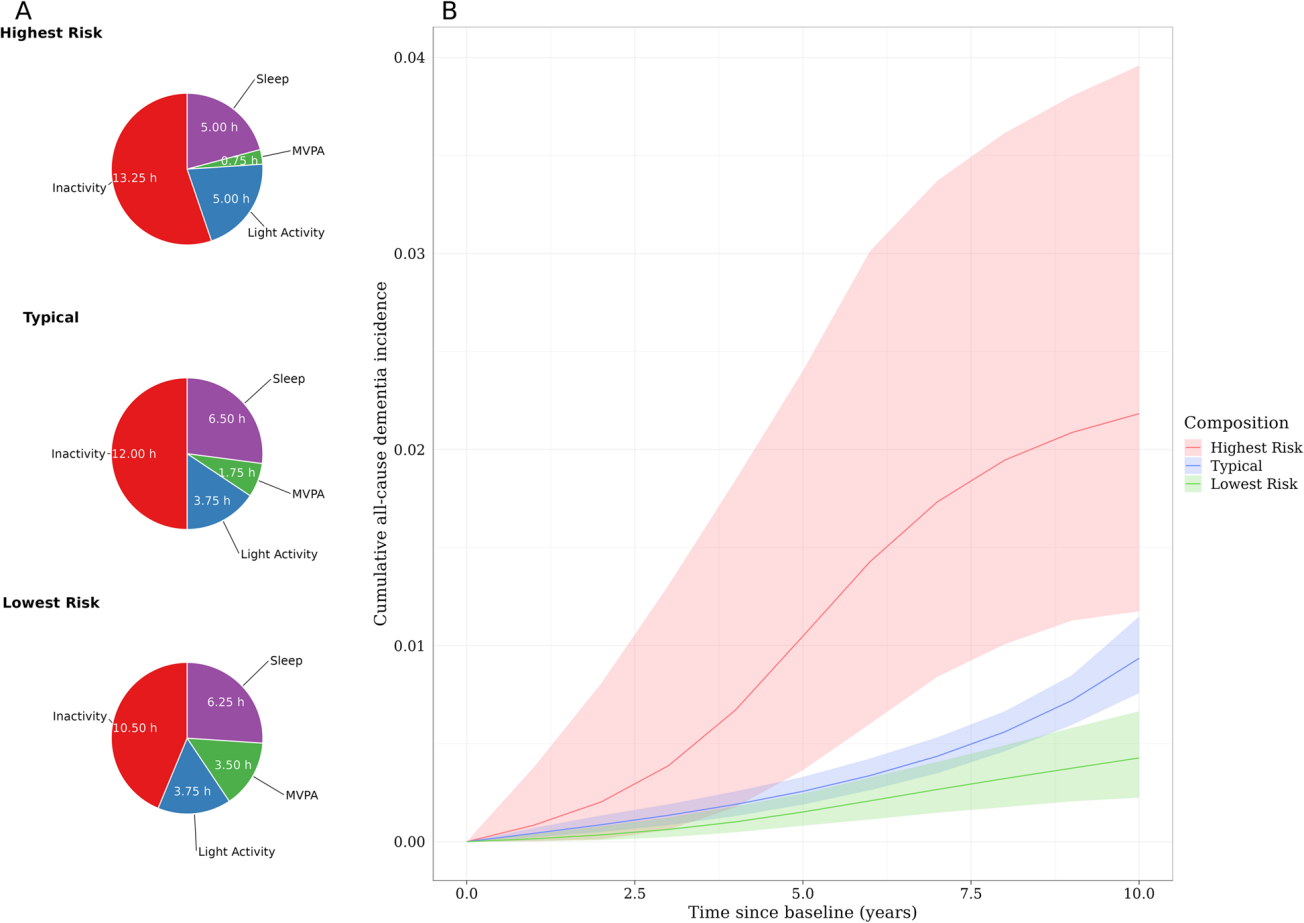
Cumulative all-cause dementia incidence for “highest risk,” “typical,” and “lowest risk” time-use compositions. **A** The constituents of the “highest risk,” “typical,” and “lowest risk” 24-h compositions. The composition that was associated with the lowest estimated dementia risk by the end of follow-up, which we refer to as the “lowest risk” composition, was represented by 10.5 h of inactivity, 6.25 h of sleep, 3.5 h of MVPA, and 3.75 h of light activity (per day, respectively). The “highest risk” composition associated with the highest estimated dementia risk was represented by 13.25 h of inactivity, 5.0 h of sleep, 0.75 h of MVPA, and 5 h of light activity. The “typical” composition was represented by 12 h of inactivity, 6.5 h of sleep, 1.75 h of MVPA, and 3.75 h of light activity. **B** The cumulative all-cause dementia incidence for each composition. MVPA, moderate to vigorous physical activity

Figure 2 plots the cumulative incidence of dementia for the estimated “highest risk,” “typical,” and “lowest risk” 24-h compositions. The highest risk composition diverged from the typical risk composition in the early period of follow-up. In comparison, the risks of dementia in the typical and lowest risk compositions were more similar and only diverged slightly in the 8th–9th year of follow-up. Notably, the minimal risk composition included > 3 h of MVPA. While this composition lies within the range of the sample data, this result should be interpreted in the context that these compositions are specifically based on wrist-worn accelerometry measures, which tend toward higher MVPA estimates compared to other forms of measurement (e.g., self-report) [31].

### MRI endophenotypes

**Fig. 3:**
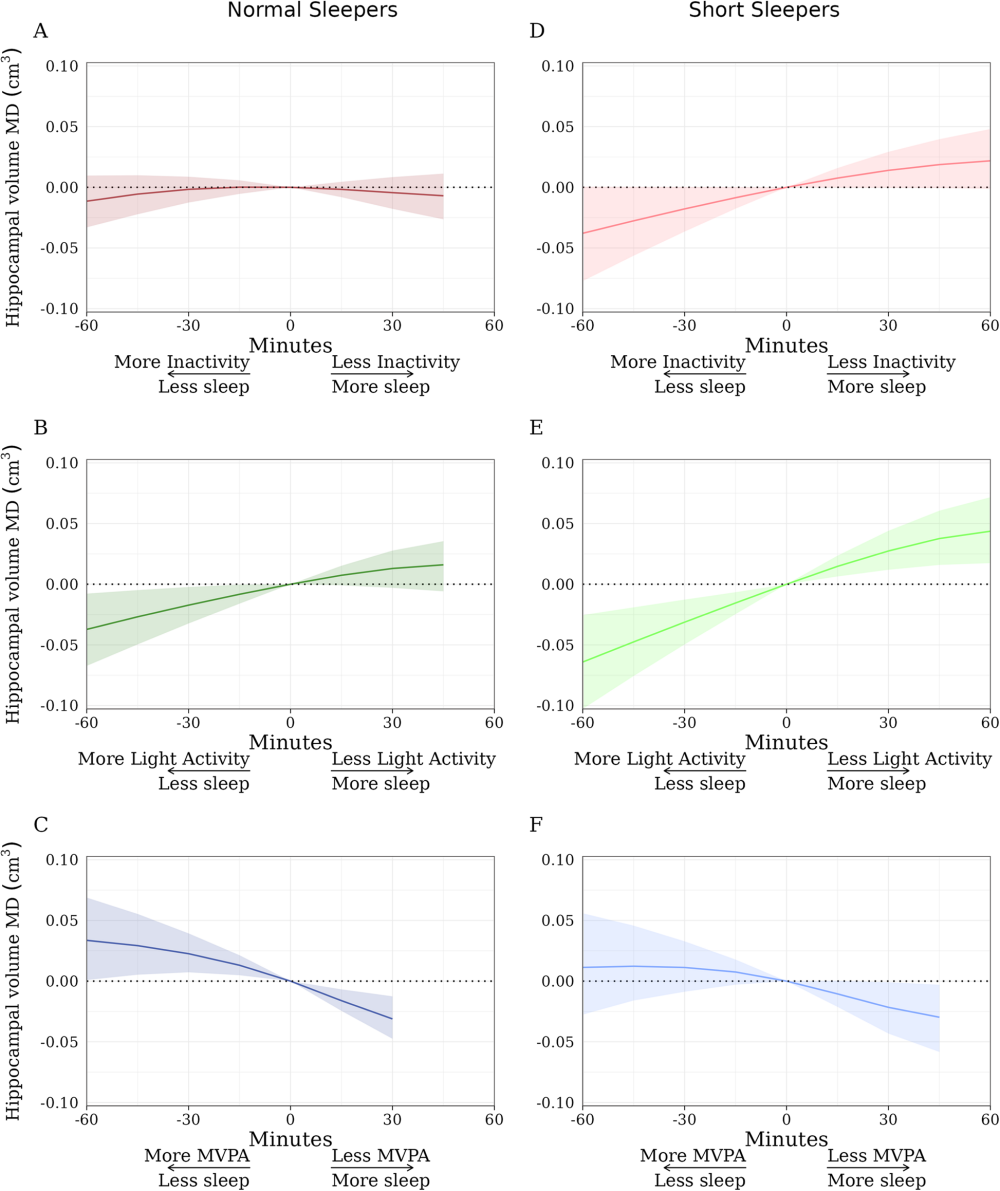
Time-use substitutions and hippocampal volume for normal (**a**, **b**, and **c**) and short sleepers (panels **d**,** e**, and **f**). MVPA = moderate to vigorous physical activity. Normal sleepers are defined as persons with ≥ 6 h of sleep (0.1% of samples had > 9 h of sleep). Short sleepers are defined as persons with < 6 h of sleep

There were 15,338 individuals with MRI data available. The association of the time-use substitutions with hippocampal volume is displayed in Fig. 3. In normal sleepers, replacing MVPA with sleep was associated with smaller hippocampal volume (e.g., 30 min; mean difference [MD], − 0.03 cm^3^; 95% CI, − 0.05, − 0.01), while replacing sleep with MVPA was associated with little change in hippocampal volume (e.g., 30 min; MD, 0.02 cm^3^; 95% CI, 0.01, 0.04). Replacing sleep with light activity was associated with smaller hippocampal volume (e.g., 30 min; MD, − 0.02 cm^3^; 95% CI, −0.03, 0.00). The pattern was similar for short sleepers (Fig. 3) (e.g., replacing MVPA with sleep was associated with smaller hippocampal volume; 30 min; MD, − 0.02 cm^3^; 95% CI, − 0.04, − 0.00) and other volumetric outcomes, including total brain volume, grey matter volume, white matter volume, and log white matter hyperintensities (Additional file 1, Figures S4-7, respectively).

**Fig. 4:**
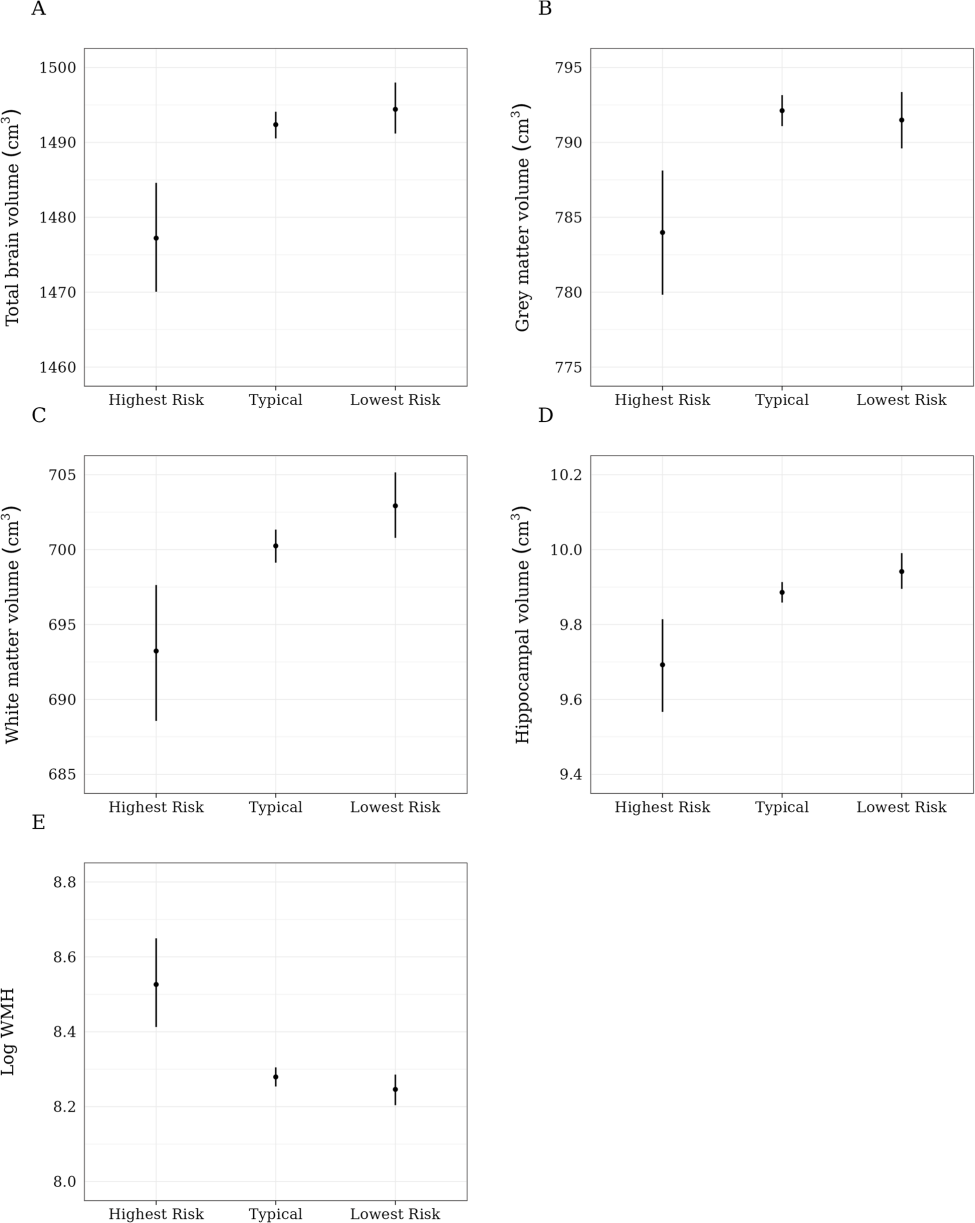
Brain MRI outcomes for “highest risk,” “typical,” and “lowest risk” time-use compositions. The highest risk, typical, and lowest risk compositions are those described in Fig. 2. The composition that was associated with the lowest estimated dementia risk by the end of follow-up, which we refer to as the “lowest risk” composition, was represented by 10.5 h of inactivity, 6.25 h of sleep, 3.5 h of MVPA, and 3.75 h of light activity (per day, respectively). The “highest risk” composition associated with the highest estimated dementia risk was represented by 13.25 h of inactivity, 5.0 h of sleep, 0.75 h of MVPA, and 5 h of light activity. The “typical” composition was represented by 12 h of inactivity, 6.5 h of sleep, 1.75 h of MVPA, and 3.75 h of light activity

Estimated MRI endophenotypes for the “highest risk,” “typical,” and “lowest risk” compositions are displayed in Fig. 4. Brain volumes tended to be similar between the “lowest risk” and “typical” compositions and lower for the “highest risk” composition. Similarly, the log of white matter hyperintensities was similar between the “lowest risk” and “typical” compositions but higher for the “highest risk” composition.

### Sensitivity analyses

The main time use substitution results for dementia were not meaningfully different after adjusting for sleep fragmentation, chronic disease, and chronic disease risk factor variables, truncating the first three years of follow-up, or after adjusting for selective participation in the UK Biobank (Additional file 1, Figure S8 and Figure S9).

## Discussion

This study examined how sleep and physical activity trade-offs relate to brain health and dementia risk. We found that dementia risk may depend on the nature of the increasing and decreasing behavior and baseline sleep levels. In short sleepers, increasing sleep duration was associated with a reduction in dementia risk as long as the behavior being substituted was not MVPA. In normal sleepers, the association of increasing sleep duration was entirely dependent on the behavior being substituted; dementia risk decreased, increased, or remained stable when substituting out light activity, MVPA, or inactivity, respectively. Findings were similar when using brain volumes as outcomes in a subset with MRI. We also identified the most and least favorable combinations of 24-h behaviors for dementia risk by the end of follow-up. Individuals with very short sleep, high inactivity, and low MVPA had the highest rates of dementia and evidence of accelerated brain aging on MRI. Overall, these results provide preliminary insights into potential behavior changes that could be targeted in interventions or guidelines to enhance brain health and prevent dementia.

Short sleep duration has been linked to poorer cognition [32–34], lower brain volumes [35], and increased dementia risk [3]. Thus, increasing sleep duration amongst short sleepers may help mitigate dementia risk. Here, we provide evidence of the specific behaviors to target in those with short sleep, namely inactivity and light activity. For those with short sleep duration, increasing sleep by 30 min instead of engaging in inactivity or light activity was associated with a 9% and 19% reduction in dementia risk, respectively. Though counterintuitive, increasing sleep at the expense of light activity rather than inactivity was more advantageous. Interestingly, others have suggested a benefit of sedentary time on cognition [36, 37]. Such results may be explained by differences in cognitive load during these daytime behaviors. Some inactivity time may be spent engaging in cognitively stimulating (e.g., writing, reading) or social activities [38]. In contrast, light activity may involve less challenging cognitive tasks (e.g., housework). Accordingly, in some people, replacing time spent inactive may reduce engagement in cognitive and social activities tied to a lower risk of dementia [39]. Of note, accelerometry does not allow inferences about cognitive load, and these explanations are largely speculative. Future studies delineating the type of cognitive activity co-occurring with inactivity and light activity would be beneficial to determine if this is the case.

Like short sleep, lower MVPA levels or self-report leisure time physical activity have been associated with higher dementia risk [6, 7]. Previous studies have demonstrated that replacing 30 min/day of sleep with the equivalent time spent in MVPA was associated with better cognition in participants with self-report sleep duration > 7 h/night [40]. Using self-report measures, others have shown that replacing sedentary behavior with physical activity is associated with decreased dementia risk in the UKB [11]. Our study extends these findings with objective assessments and a greater range of activity substitutions. We show that increasing MVPA by 30 min at the expense of sleep in those with normal sleep duration was associated with 24% lower dementia risk. So, waking up 30 min early to exercise may benefit the brain. *But* this same effect does not apply to short sleepers; the benefits of MVPA on dementia risk depend on whether one gets adequate sleep (in this case, ≥ 6 h). These results are consistent with findings from the English Longitudinal Study on Aging that found that the benefits of physical activity on cognitive decline were blunted in older adults who self-reported short (< 6 h) versus normal (6–8 h) sleep duration, with short sleepers having a more rapid 10-year decline [41]. These findings suggest that the neuroprotective effects of MVPA may not fully overcome the detrimental effects of short sleep.

This study also explored the highest risk, typical, and lowest risk 24-h activity compositions related to dementia risk. Those with high amounts of inactivity combined with low amounts of sleep and MVPA had the highest dementia risk and evidence of accelerated brain aging. These compositions align well with current knowledge of these behaviors and dementia risk [6, 7, 42]. Our data also suggest that transitioning people from the “highest risk” to the “typical” composition may have a far greater impact on dementia prevention as compared to transitioning people from the “typical” to the “lowest risk” composition.

We acknowledge that the “lowest risk” composition of achieving 3.3 h/day of MVPA appears relatively high, given that the current WHO recommendations are 150–300 min/week to achieve health benefits. Note that wrist-worn accelerometry can overestimate MVPA compared to hip-worn devices [43] and self-report measures. It has been estimated that 150 min/week of self-reported MVPA equals ~ 1000 min/week of device-assessed MVPA [31]. As wrist-worn devices with inbuilt activity trackers become more popular amongst the general public and are more easily implemented in large studies, our analysis captures measures of MVPA that may better reflect emerging societal and research trends. Nonetheless, the unique substitutions in this study overcome these nuances and are more applicable in providing a framework for personalized interventions, irrespective of ideal behavior targets. That is, an increase in MVPA by 15 min is more achievable than simply aiming for the lowest risk composition.

Since common causes of dementia, such as AD, have a long preclinical phase and the follow-up duration in this study was limited to 8.2 years, we do not provide direct evidence that optimizing 24-h time-use patterns can directly prevent dementia. However, it remains possible that, in the presence of early AD, optimizing 24-h behaviors may provide several benefits to the brain and cognition, which could theoretically help delay dementia. For example, glymphatic clearance of AD proteins (amyloid-β and tau) is maximal during sleep and thought to be coupled to slow wave sleep [1]. Shorter sleep duration may impair the clearance of these waste products, accelerating AD progression [1, 2]. Short sleep can also increase blood pressure [44] and inflammatory processes [45], potentially explaining links between short sleep and vascular brain injury and brain atrophy [46]. MVPA also has neuroprotective effects. That is, physical activity is thought to induce favorable alterations in cerebral blood flow, augment cognitive reserve via neuroplasticity processes, facilitate glymphatic clearance of amyloid-β, and reduce other dementia risk factors such as cardiovascular disease and stress [47]. Thus, regardless of the factors that initiate AD, optimizing 24-h behaviors could provide benefits to brain health, especially in high-risk populations such as persons at high risk of AD dementia.

### Limitations

A primary limitation of this observational analysis is the potential for confounding, especially reverse causation. Several forms of dementia have a long preclinical phase, longer in many cases than the median follow-up duration for dementia of 8.2 years in our sample [48]. The observed behavioral compositions may consequently have been influenced by preclinical disease. Though impossible to rule out, we didn’t find strong evidence of reverse causation. Truncating the first three years of follow-up (during which reverse causation would be expected to be strongest) did not substantively alter the estimated risk ratios. Another limitation included the timing of when covariates were captured. Where possible, we included covariates available at the time of accelerometry. However, some of our covariates, including income, retirement status, shift work and smoking status, were only available at study entry (i.e., at the baseline UKB assessment). We also recognize that our categorization of time use into, for instance, inactivity, does not distinguish sitting and standing, nor varied behaviors that may occur as part of those categories (e.g., reading, socializing, or watching television), some of which may plausibly be related to dementia risk in different ways. Furthermore, as we did not have longitudinal data on 24-h behaviors, the time use substitution that we investigated do not constitute well-defined interventions (i.e., with a clear start time and duration) [49, 50]. We also could not determine whether participants were chronic short, normal, or long sleepers. Although this paper presents an important first step, future randomized trials or observational studies could be designed to clarify the effect of substitutions with a defined time course. Due to the lack of a sleep diary in the UKB, we were unable to identify daytime naps occurring outside the main sleep bout. Napping has been associated with dementia risk [51], and it would be interesting to consider napping in future studies employing the CoDA framework. UKB does not include polysomnographic sleep measures meaning that we were unable to accurately adjust for sleep-related pathologies such as obstructive sleep apnea or restless leg syndrome. Finally, those who participated in the UKB cohort tend to be healthier and of higher socioeconomic status than general UK population, leading to potential issues with external validity [13]. Nevertheless, we did not find meaningfully different results in our sensitivity analysis, which at least partly corrected for selective participation in the UKB.

## Conclusions

Our data suggests that strategies for dementia risk reduction may require a tailored approach that assesses all 24-h behaviors and targets those behaviors that are amenable to have optimal effect. We found that, depending on whether individuals had short or normal sleep duration, reallocating time from inactivity or light activity to sleep was favorably associated with brain aging and dementia risk. A similar pattern was observed for reallocating time from sleep to MVPA, showing stronger benefits in those with normal sleep and weaker effects in short sleepers. These data indicate that there could be some flexibility in setting behavior change goals to enable an achievable behavior change for the individual. For example, increasing MVPA would be an obvious choice to improve brain health, but getting enough MVPA could be challenging in some populations. Our data show that even an increase in sleep by 30 min/day (in place of light activity) may benefit these groups where reaching physical activity targets may be challenging.

This study supports the contention that combination therapies, targeting all 24-h behaviors, could be the next best step forward for lifestyle risk reduction for dementia. However, future intervention trials are required to confirm whether this approach would be effective.

## Supplementary Information

Additional file 1. Sleep and Physical Activity Trade-offs and Dementia Risk: A Prospective Cohort Study in UK Biobank Participants. Additional file 1 contains eMethods for procedure of estimating dementia risks. Table S1 – Estimated dementia risk ratios for time use substitutions. Figure S1 - Flow chart for sample selection. Figure S2 - Causal directed acyclic graph. Figure S3 - All-cause dementia risk ratiosfor time-use substitutions for long sleepers. Figures S4-7 - Time-use substitutions for MRI brain volumes. Figure S8 -Dementia sensitivity analyses for normal sleepers. Figure S9 - Dementia sensitivity analyses for short sleepers

## Data Availability

This research was conducted using the UK Biobank data under application number 70607. Data can be accessed following an approved application, which can be completed via the UKB website: [https://www.ukbiobank.ac.uk/]. All analysis code is available at the project GitHub repository (https://github.com/BeaudanBrown/coda-dementia).

## Abbreviations

MVPA: Moderate to vigorous physical activity
UKB: United Kingdom Biobank
CoDA: Compositional data analysis
AD: Alzheimer’s disease
Q1: First quartile
Q3: Third quartile
SES: Socioeconomic status
MRI: Magnetic resonance imaging
IDP: Imaging-derived phenotypes
APOE ε4: Apolipoprotein E epsilon 4
ENMO: Euclidean Distance Minus One
ICD-10: International Classification of Diseases, 10th edition
ILR: Isometric log ratio
fMRI: Functional magnetic resonance imaging
BMI: Body mass index
CVD: Cardiovascular disease
RR: Risk ratio
95% CI: 95% Confidence intervals
WASO: Wake after sleep onset
