# Supplementary material for: Sleep and physical activity trade-offs and dementia risk: a prospective cohort study in UK Biobank participants
Source: BMC Med. 2025 Dec 3;24:11. doi: 10.1186/s12916-025-04536-7 (PMC12781825; doi:10.1186/s12916-025-04536-7)
Supplement: Supplementary file 1 — Additional file 1. Sleep and Physical Activity Trade-offs and Dementia Risk: A Prospective Cohort Study in UK Biobank Participants. Additional file 1 contains eMethods for procedure of estimating dementia risks. Table S1 – Estimated dementia risk ratios for time use substitutions. Figure S1 - Flow chart for sample selection. Figure S2 - Causal directed acyclic graph. Figure S3 - All-cause dementia risk ratiosfor time-use substitutions for long sleepers. Figures S4-7 - Time-use substitutions for MRI brain volumes. Figure S8 -Dementia sensitivity analyses for normal sleepers. Figure S9 - Dementia sensitivity analyses for short sleepers [file 12916_2025_4536_MOESM1_ESM.docx]

**Additional File 1**

**Yiallourou, Cribb, Campbell-Brown, et al. Sleep and Physical Activity Trade-offs and Dementia Risk: A Prospective Cohort Study in UK Biobank Participants**

Correspondence to: matthewpase@monash.edu

**Contents**

eMethods. Procedure for estimating dementia risk ratios for isotemporal substitutions

eMethods. Procedure for estimating ideal, typical, and worst composition

Table S1. Estimated dementia risk ratios for time use substitutions

Figure S1. Sample selection

Figure S2. Causal directed acyclic graph

Figure S3. All-cause dementia risk ratio (and 95% confidence interval) for time-use substitutions for long sleepers

Figure S4. Time-use substitutions and total brain volume for normal and short sleepers.

Figure S5. Time-use substitutions for grey matter volume for normal and short sleepers.

Figure S6. Time-use substitutions for white matter volume for normal and short sleepers.

Figure S7. Time-use substitutions for log white matter hyperintensities for normal and short sleepers.

Figure S8. Dementia sensitivity analyses for normal sleepers

eFigure 9. Dementia sensitivity analyses for short sleepers

eReferences.

**eMethods.**

**Procedure for estimating dementia risk ratios for isotemporal substitutions**

Risk ratios for isotemporal substitutions were estimated using the following procedure.

1. *Fit pooled logistic regression*

Pooled logistic models (parametric models which approximate the parameters of the Cox proportional hazards model) were fitted for dementia and death, including ILR coordinates and covariates as predictors. For these models, follow-up was divided into discrete chunks, 1 year in length, such that each participant had as many rows of data as they had discrete follow-up intervals, ending either at the time of dementia, death, or end of study follow-up. Follow-up time was modeled with a restricted cubic spline with 5 knots. Time was used as the timescale. All continuous covariates were modeled with restricted cubic splines (knots at the 10^th^, 50^th^, and 90^th^ percentiles) to allow for departures from linearity. Product terms between ILR coordinates and time, ILR coordinates and key covariates, key covariates and time, and key covariates and key covariates were included.

1. *Estimating dementia risk*

For each subgroup (i.e., short or normal sleepers), a new dataset including all participants over all follow-up times was created. The hazards of dementia and death over follow-up were estimated for this dataset, without any intervention applied to the observed time use composition, based on the fitted pooled logistic model fitted in step 1. Dementia risk, accounting for the competing event of death, was estimated as a function of these hazards. This provided the “reference” risk (i.e. the risk under no intervention on time use composition). We then modified this dataset by applying a series of isotemporal “substitutions” to the observed time use composition (e.g., adding 30 minutes of sleep at the expense of inactivity), and estimated dementia risk under each of these perturbed compositions in the same manner.

1. *Risk ratios and confidence intervals*

The ratios of dementia risk by the end of follow-up (approximately 9.5 years from baseline) under each substitution relative to the reference risk (i.e., under no substitution) were calculated. Nonparametric bootstrapping (500 samples) with nested single imputation of missing data was used to obtain percentile-based 95% confidence intervals.

**Procedure for estimating lowest risk, typical, and highest risk compositions**

To estimate the lowest risk, typical, and highest risk compositions, the following steps were performed:

1. We estimated the multivariate density function, using a kernel density estimator, of the isometric log ratio transformed composition data. We then estimated the density for each composition in the dataset and calculated the 5th percentile, representing the threshold for “low” density (i.e., relatively rare compositions).
2. We created a grid of synthetic compositions (in 15-minute steps) covering all possible compositions, with each component (e.g., sleep time, MVPA time) of the composition restricted to the 2.5th and 97.5th quantiles of the sample data. The univariate limits for the synthetic compositions were 3.25 to 9 hours for sleep, 0.5 to 6.25 hours for MVPA, 1.5 to 8.5 hours for light activity, and 6 to 17.5 hours for inactivity. Each member of the grid of compositions was then passed into the density function estimated in (1) and any composition with density below the “low” density threshold was excluded, thereby removing compositions that would be rarely observed from consideration. After filtering, 13,624 synthetic compositions were retained.
3. We split the data randomly into two equally sized folds
4. In the first (“training”) fold, we estimated dementia risk for each synthetic composition using the g-computation procedure described in the main text. The synthetic composition returning the lowest and highest estimated dementia risks were termed the “lowest risk” and “highest risk” compositions, respectively. The “typical” composition was that with the highest estimated density.
5. In the second (“testing”) fold the cumulative incidence of dementia for each of the lowest risk, highest risk, and typical compositions from step (4) was then estimated using the g-computation procedure described in the main text.

**Table S1: Estimated dementia risk ratios for time use substitutions**

| **Subgroup** | **Change in sleep time (minutes)** | **Sleep/inactivity substitution** | | **Sleep/light activity substitution** | | **Sleep/MVPA substitution** | |
| --- | --- | --- | --- | --- | --- | --- | --- |
|  |  | *Risk ratio (95% CI)* | *% receiving substitution* | *Risk ratio (95% CI)* | *% receiving substitution* | *Risk ratio (95% CI)* | *% receiving substitution* |
| Normal sleepers | -60 | 1.06 (0.98, 1.16) | 99.7 | 1.45 (1.26, 1.64) | 100 | 0.66 (0.52, 0.80) | 100 |
|  | -45 | 1.03 (0.96, 1.10) | 99.6 | 1.30 (1.17, 1.43) | 100 | 0.70 (0.59, 0.80) | 100 |
|  | -30 | 1.00 (0.96, 1.06) | 99.3 | 1.18 (1.10, 1.26) | 99.8 | 0.76 (0.69, 0.83) | 99.9 |
|  | -15 | 1.00 (0.97, 1.02) | 98.7 | 1.08 (1.04, 1.12) | 99.3 | 0.86 (0.82, 0.90) | 99.5 |
|  | 15 | 1.02 (0.99, 1.05) | 95.4 | 0.94 (0.90, 0.97) | 94.5 | 1.17 (1.11, 1.21) | 93.3 |
|  | 30 | 1.04 (0.98, 1.10) | 91.1 | 0.89 (0.83, 0.96) | 87.6 | 1.30 (1.21, 1.40) | 83.5 |
|  | 45 | 1.07 (0.98, 1.16) | 83.8 | 0.88 (0.80, 0.96) | 75.9 | NA | 68.1 |
|  | 60 | NA | 72.3 | NA | 59.2 | NA | 49.4 |
| Short sleepers | -60 | 1.32 (1.17, 1.47) | 99.9 | 1.71 (1.49, 1.97) | 100 | 0.84 (0.68, 1.03) | 100 |
|  | -45 | 1.22 (1.12, 1.32) | 99.9 | 1.48 (1.34, 1.64) | 100 | 0.84 (0.73, 0.97) | 100 |
|  | -30 | 1.13 (1.07, 1.20) | 99.8 | 1.29 (1.21, 1.38) | 100 | 0.86 (0.79, 0.95) | 100 |
|  | -15 | 1.06 (1.03, 1.09) | 99.8 | 1.13 (1.09, 1.17) | 99.7 | 0.91 (0.87, 0.96) | 100 |
|  | 15 | 0.95 (0.93, 0.97) | 99.5 | 0.89 (0.86, 0.92) | 97.2 | 1.09 (1.05, 1.14) | 93.5 |
|  | 30 | 0.91 (0.86, 0.95) | 99.3 | 0.81 (0.76, 0.87) | 93.5 | 1.17 (1.09, 1.26) | 85.3 |
|  | 45 | 0.87 (0.82, 0.93) | 99 | 0.77 (0.70, 0.84) | 87.6 | NA | 73.8 |
|  | 60 | 0.85 (0.78, 0.92) | 98.7 | 0.75 (0.67, 0.83) | 78.9 | NA | 59.8 |

Full UKB sample (accessed March 2024): n = 502,180

Did not participate in accelerometry sub-study: n = 398,553

Accelerometry data unreliable using UKB criteria (inadequate wear time, poor calibration): n = 9,313

**Incident dementia analysis**

Further filtering (GGIR quality control, filtering extreme quantiles): n= 5,990

Valid accelerometry data available: n = 88,324

Prevalent dementia or other severe neurological disease (n = 885)

Final incident dementia analysis sample: n = 87,490

MRI data unavailable: n = 71,156

MRI study before accelerometry: n = 1,154

**MRI analysis**

Final MRI analysis sample: n = 15,180

**Figure S1. Sample selection.** MRI = magnetic resonance imaging. Severe neurological disease included Parkinson's disease, motor neuron disease, cerebral palsy, brain abscess, multiple sclerosis, or myasthenia gravis


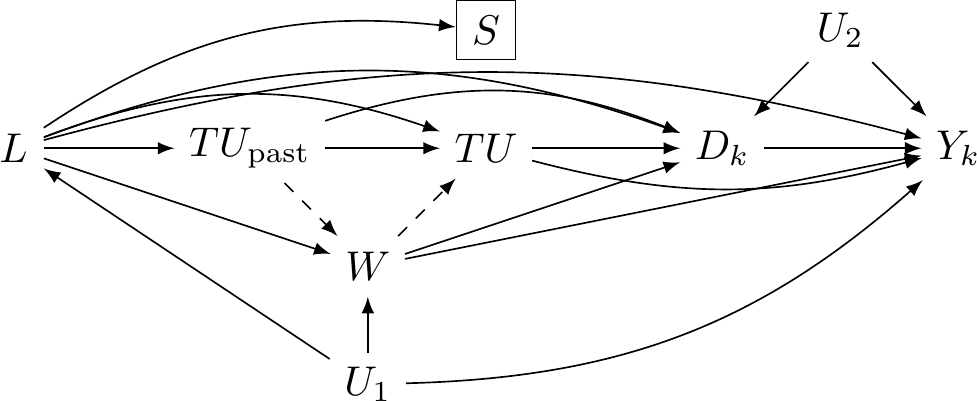


**Figure S2**. **Causal directed acyclic graph.** $TU$ represents time use at baseline and $TU_{past}$ represents time use before the study baseline (unmeasured). $L$ represents the set of confounders, measured at baseline, which are presumed to cause, but not be caused by, 24-hour behaviors. These are: age, sex, education, ethnicity, APOE-e4 genotype, household income, antidepressant, antipsychotic, and sedative medication use, retirement status, and shift work. $W$ is the set of variables that *may* lie on the causal path between 24-hour behaviors and dementia incidence. Based on a consideration of the existing evidence and discussion and consensus within our group, we determined these variables to be history of cardiovascular disease, diabetes, BMI, blood pressure medication, systolic blood pressure, and sickness or disability (employment category). $S$ represents selection into the accelerometry sample, caused by the variables in $L$. $Y_{k}$ is dementia occurrence at time $k$ of follow-up. $D_{k}$ represents death at time $k$. $U_{1}$ is the set of unmeasured shared causes of $L$, $W$ and $Y_{k}$ and $U_{2}$ is the set of unmeasured shared causes of $D_{k}$ and $Y_{k}$ (e.g., genetics, physiological dysregulation). Paths represent the direction of causal effects between nodes. Dashed lines into and out of *W* indicate paths that we are uncertain about.

Given this DAG, we are presented with two options for confounder adjustment: adjusting for the variables in $W$ or not adjusting for the variables in $W$ (in addition to adjusting for the variables in $L$). If the path from $TU_{past}$ to $W$ is present, then $W$ is on the causal path between time use and the outcome. In this case, we ought not to adjust for $W$. If the path from $W$ to $TU_{past}$ is present, then $W$ is a confounder. In this case, adjusting for $W$ will eliminate confounding at the expense of introducing bias due to adjusting for possible mediators, if the $TU_{past}$ to $W$ path exists. We present results using both adjustment strategies.

**
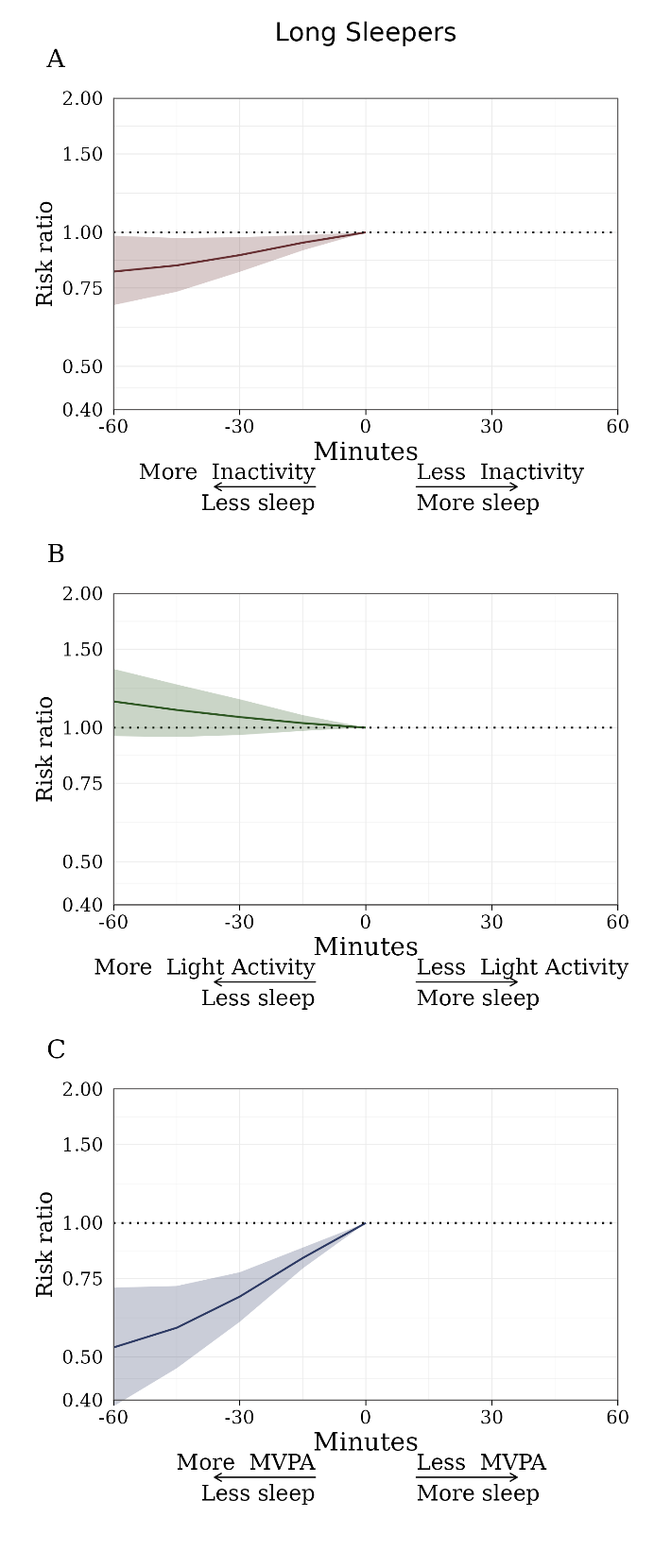
**

**Figure S3. All-cause dementia risk ratio (and 95% confidence interval) for time-use substitutions for long sleepers.** MVPA = moderate to vigorous physical activity. Long sleepers are defined as persons with >8 hours of sleep.

**
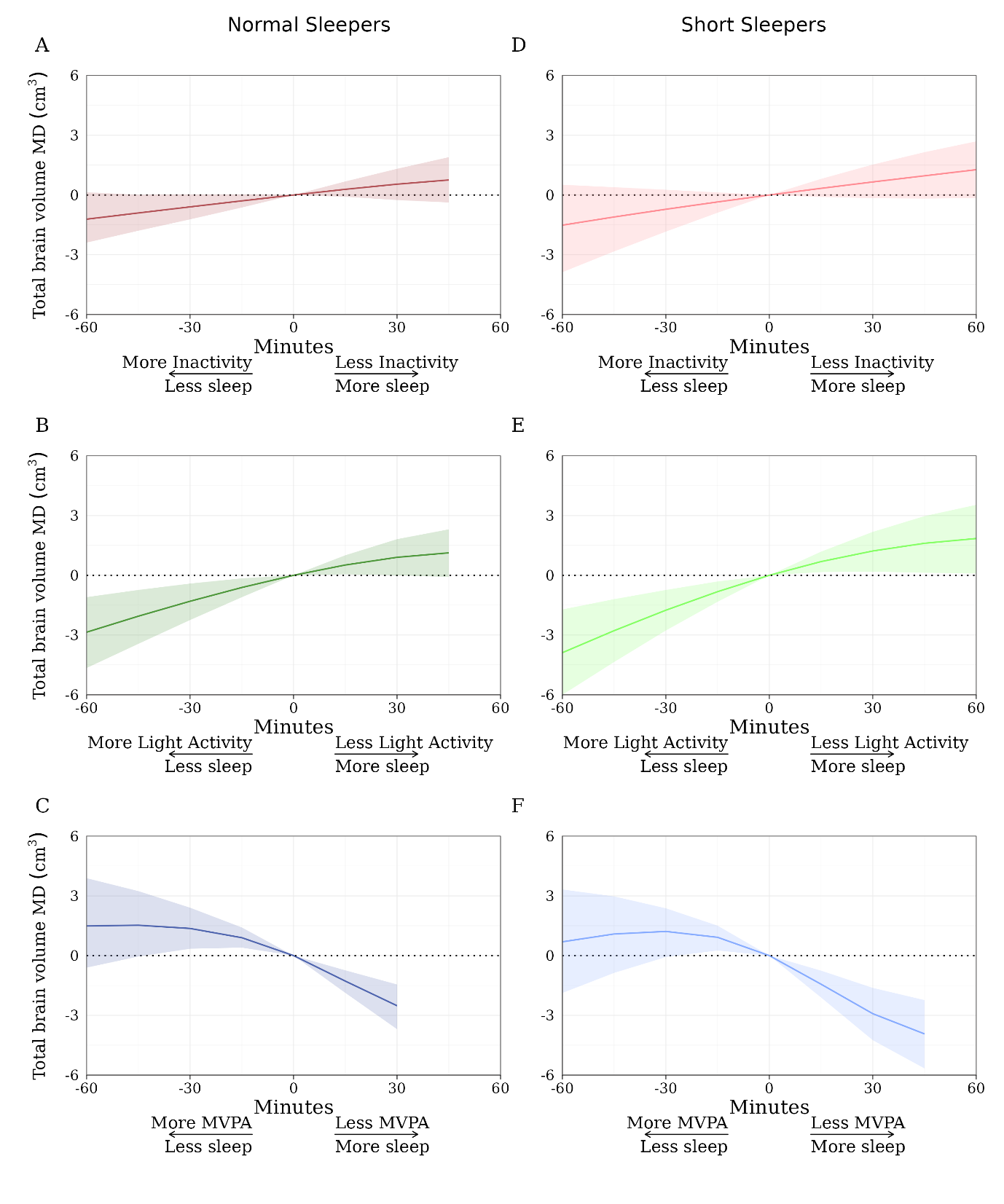
**

**Figure S~~4~~**. **Time-use substitutions & total brain volume for normal & short sleepers**. MVPA = moderate to vigorous physical activity. Normal sleepers are defined as persons with ≥6 hours and ≤9 hours of sleep. Short sleepers are defined as persons with <6 hours of sleep.

**
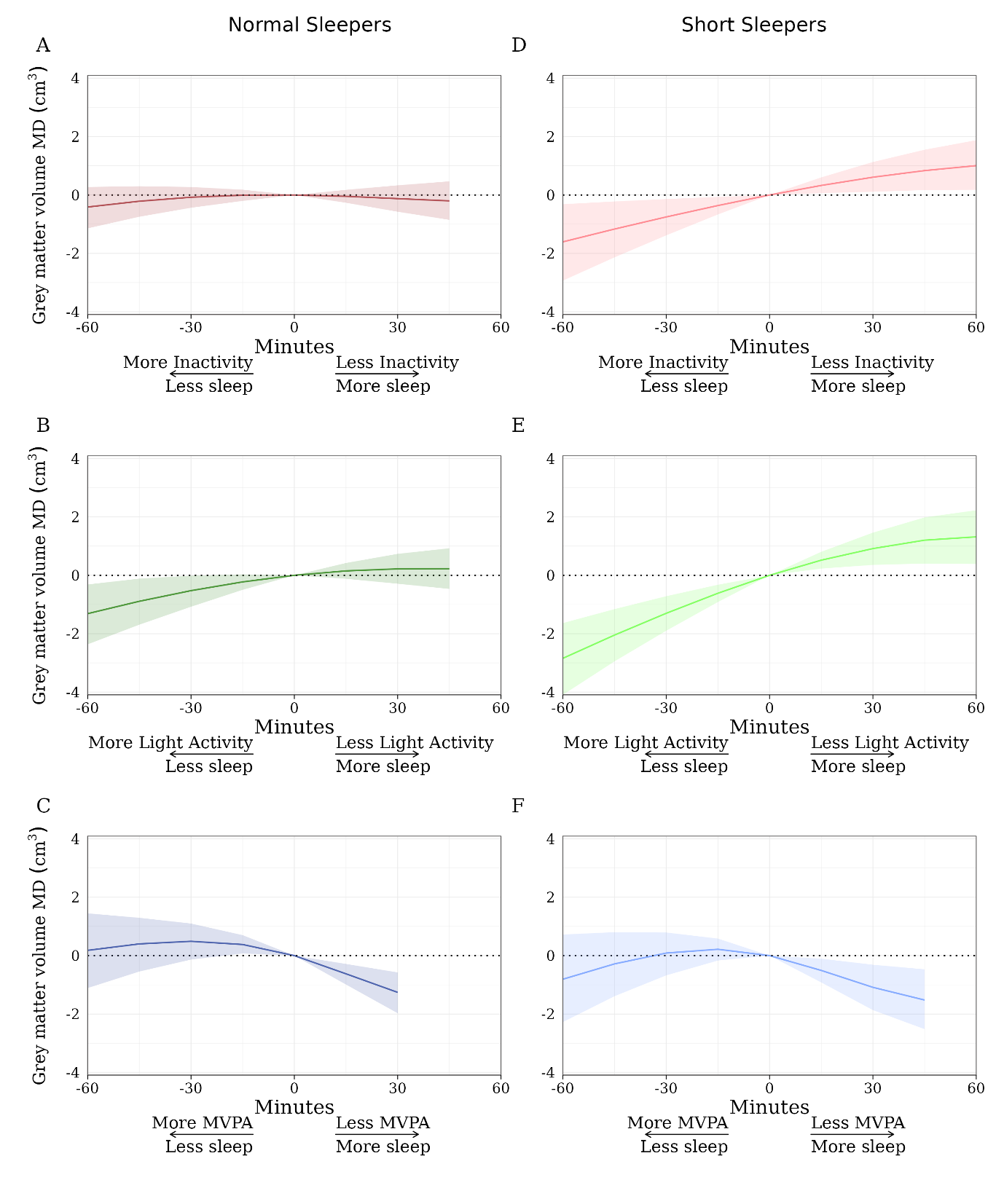
**

**Figure S5. Time-use substitutions for grey matter volume for normal & short sleepers.** MVPA = moderate to vigorous physical activity. Normal sleepers are defined as persons with ≥6 hours and ≤9 hours of sleep. Short sleepers are defined as persons with <6 hours of sleep.

**
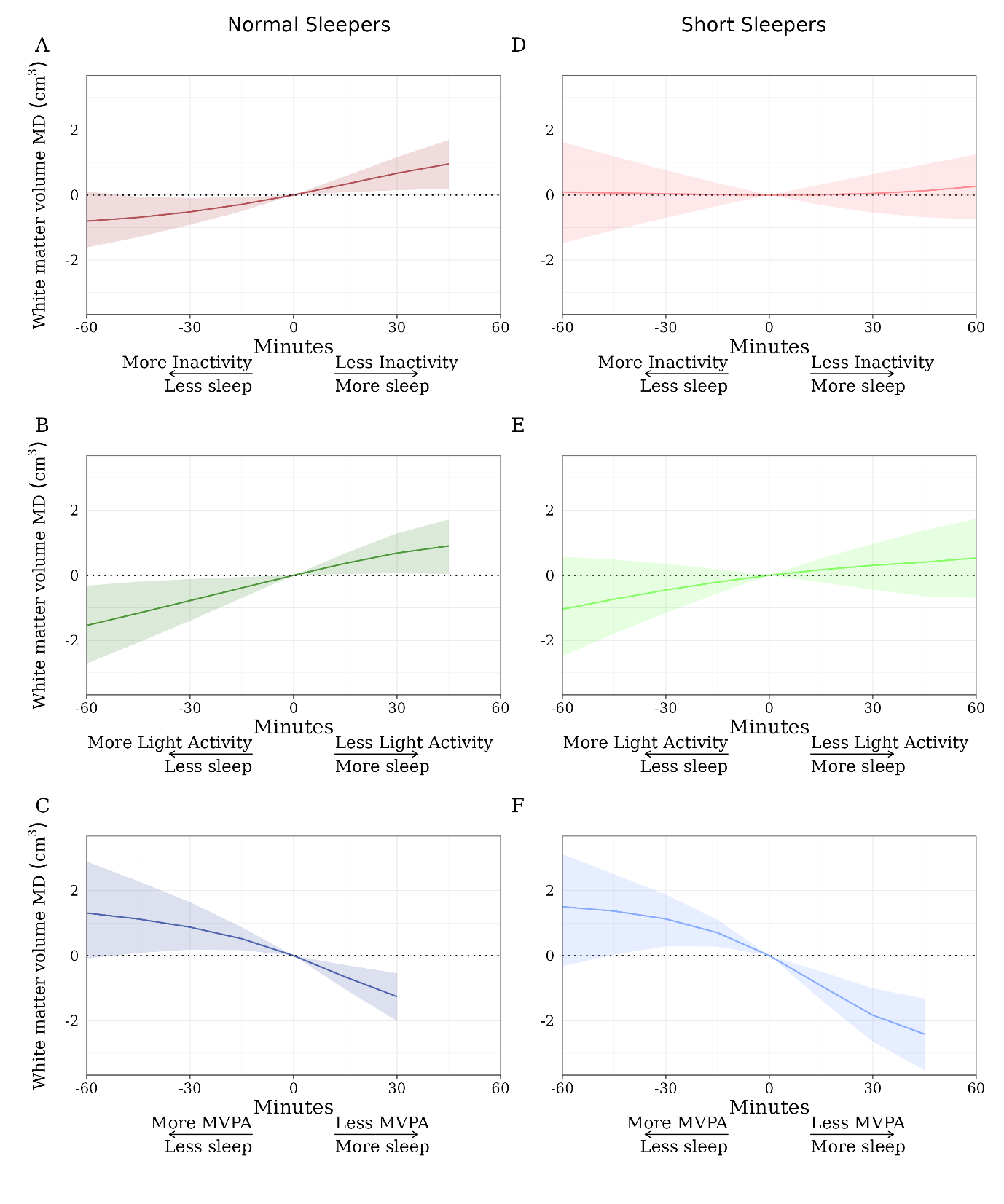
**

**Figure S6**. **Time-use substitutions for white matter volume, for normal and short sleepers.** MVPA = moderate to vigorous physical activity. Normal sleepers are defined as persons with ≥6 hours and ≤9 hours of sleep. Short sleepers are defined as persons with <6 hours of sleep.

**
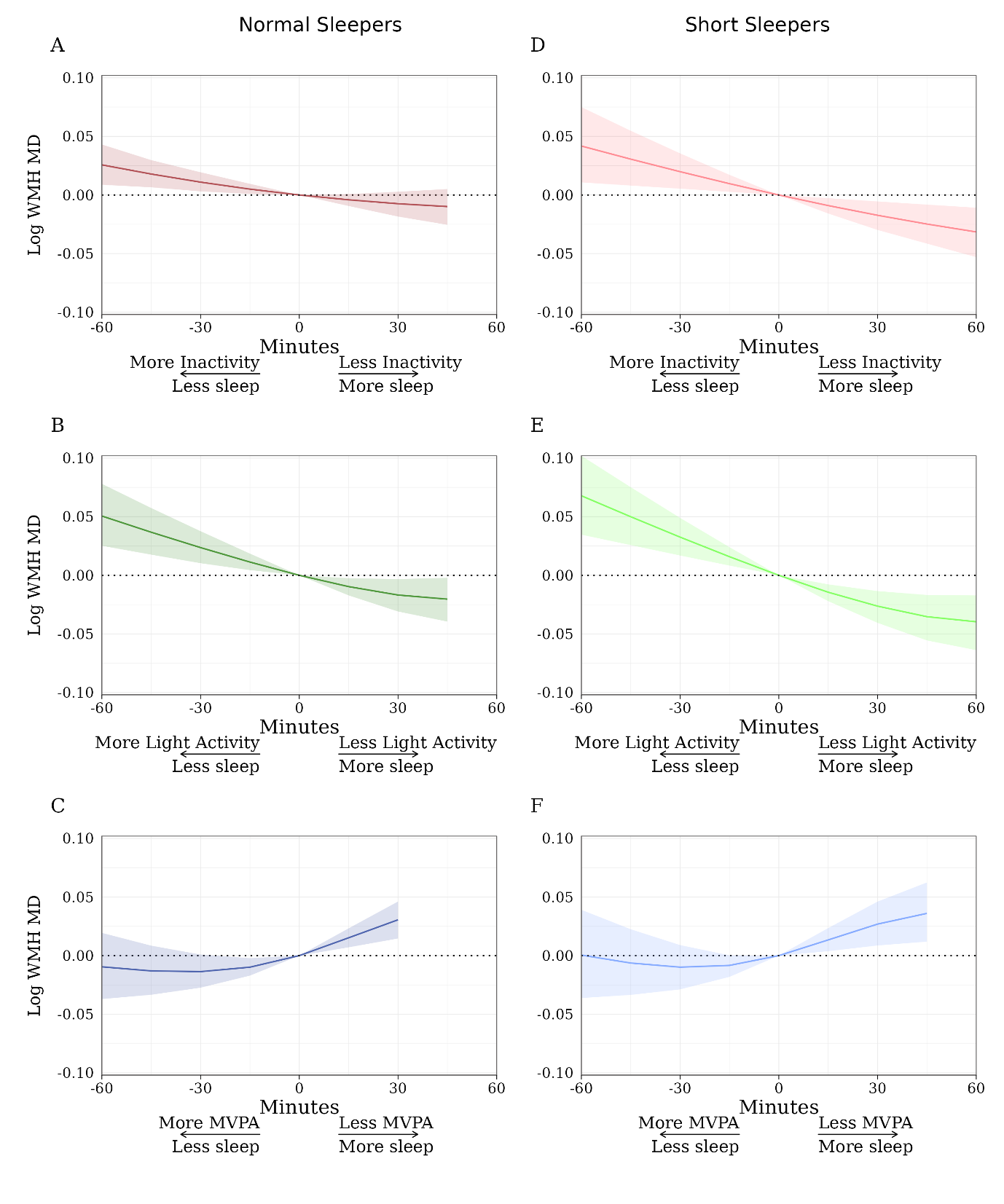
**

**Figure S7. Time-use substitutions for log white matter hyperintensities for normal and short sleepers.** MVPA = moderate to vigorous physical activity. Normal sleepers are defined as persons with ≥6 hours and ≤9 hours of sleep. Short sleepers are defined as persons with <6 hours of sleep

**
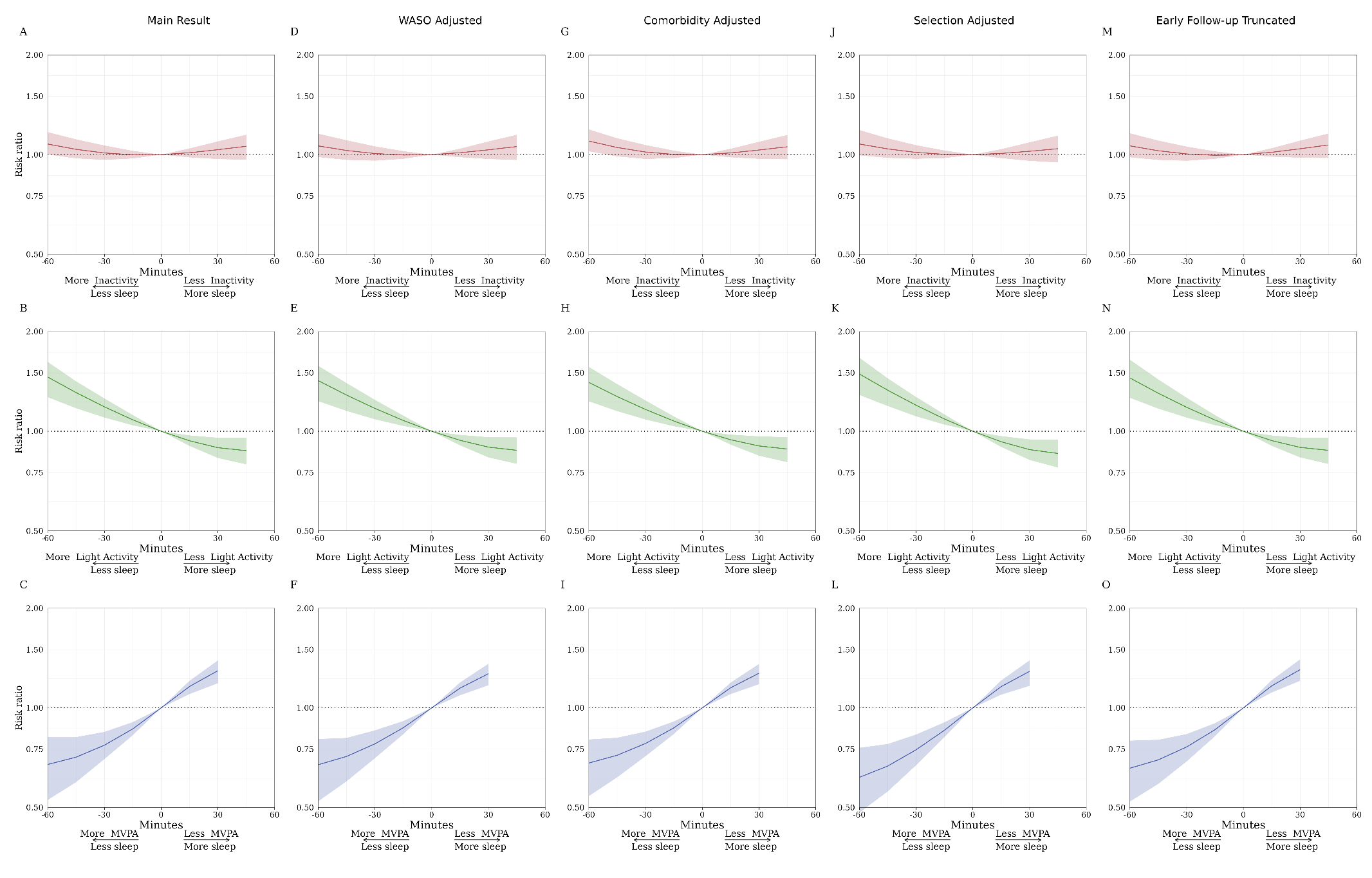
**

**Figure S8. Dementia sensitivity analyses for normal sleepers**

A to C: Primary all-cause dementia risk ratio for time-use substitutions for normal sleepers

D to F: All-cause dementia risk ratio for time-use substitutions for normal sleepers, with adjustment for the covariates outlined in the main text plus wake after sleep onset**.**

G to I: All-cause dementia risk ratio for time-use substitutions for normal sleepers, with adjustments for the covariates outlined in the main text plus disease history and disease risk factors, including sick/disabled (self-reported employment category), history of diabetes, history of cancer, history of a mental disorder, history of a nervous system disease (excluding severe neurological diseases which are excluded from the sample), history of cardiovascular disease, systolic blood pressure, blood pressure medication, and body mass index.

J to L: All-cause dementia risk ratio for time-use substitutions for normal sleepers, correcting for selective participation in the UK Biobank by standardizing to a set of representative UK population characteristics.

M to O: All-cause dementia risk ratio for time-use substitutions for normal sleepers, truncating the first three years of follow-up.

MVPA = moderate to vigorous physical activity. Normal sleepers are defined as persons with ≥6 hours and ≤9 hours of sleep.

**
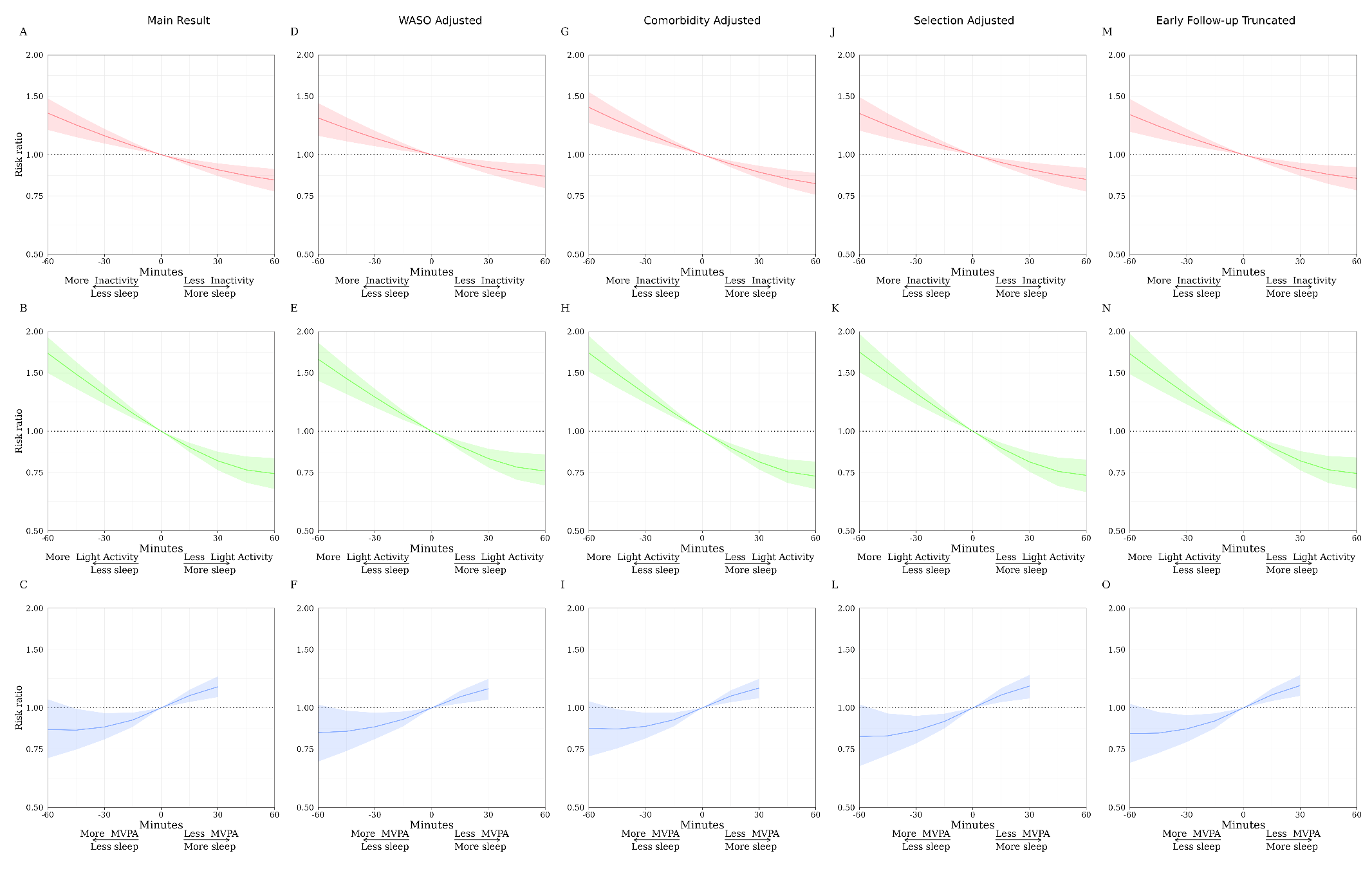
**

**Figure S9. Dementia sensitivity analyses for short sleepers**

A to C: Primary all-cause dementia risk ratio for time-use substitutions for short sleepers

D to F: All-cause dementia risk ratio for time-use substitutions for short sleepers, with adjustment for the covariates outlined in the main text plus wake after sleep onset**.**

G to I: All-cause dementia risk ratio for time-use substitutions for short sleepers, with adjustments for the covariates outlined in the main text plus disease history and disease risk factors, including sick/disabled (self-reported employment category), history of diabetes, history of cancer, history of a mental disorder, history of a nervous system disease (excluding severe neurological diseases which are excluded from the sample), history of cardiovascular disease, systolic blood pressure, blood pressure medication, and body mass index.

J to L: All-cause dementia risk ratio for time-use substitutions for short sleepers, correcting for selective participation in the UK Biobank by standardizing to a set of representative UK population characteristics.

M to O: All-cause dementia risk ratio for time-use substitutions for short sleepers, truncating the first three years of follow-up.

MVPA = moderate to vigorous physical activity. Short sleepers are defined as persons with <6 hours of sleep.
